# Maternal obesity in the ewe increases cardiac ventricular expression of glucocorticoid receptors, proinflammatory cytokines and fibrosis in adult male offspring

**DOI:** 10.1371/journal.pone.0189977

**Published:** 2017-12-21

**Authors:** Adel B. Ghnenis, John F. Odhiambo, Richard J. McCormick, Peter W. Nathanielsz, Stephen P. Ford

**Affiliations:** Center for the Study of Fetal Programming, Department of Animal Science, University of Wyoming, Laramie, WY, United States of America; University of Southampton, UNITED KINGDOM

## Abstract

Obesity during human pregnancy predisposes offspring to obesity and cardiovascular disease in postnatal life. In a sheep model of maternal overnutrition/obesity we have previously reported myocardial inflammation and fibrosis, as well as cardiac dysfunction in late term fetuses, in association with chronically elevated blood cortisol. Significant research has suggested a link between elevated glucocorticoid exposure *in utero* and hypertension and cardiovascular disease postnatally. Here we examined the effects of maternal obesity on myocardial inflammation and fibrosis of their adult offspring. Adult male offspring from control (CON) mothers fed 100% of National Research Council (NRC) recommendations (n = 6) and male offspring from obese mothers (MO) fed 150% NRC (n = 6), were put on a 12-week *ad lib*itum feeding challenge then necropsied. At necropsy, plasma cortisol and left and right ventricular thickness were markedly increased (P<0.05) in adult male MO offspring. Myocardial collagen content and collagen-crosslinking were greater (P<0.05) in MO offspring compared to CON offspring in association with increased mRNA and protein expression of glucocorticoid receptors (GR). No group difference was found in myocardial mineralocorticoids receptor (MR) protein expression. Further, mRNA expression for the proinflammatory cytokines: cluster of differentiation (CD)-68, transforming growth factor (TGF)-β1, and tumor necrosis factor (TNF)-α were increased (P < 0.05), and protein expression of CD-68, TGF-β1, and TNF-α tended to increase (P<0.10) in MO vs. CON offspring. These data provide evidence for MO-induced programming of elevated plasma cortisol and myocardial inflammation and fibrosis in adult offspring potentially through increased GR.

## Introduction

Obesity is considered a worldwide public health concern, with about one-third of adults in the US currently classified as obese [1]. Further, obesity rates of women during pregnancy are between 20% and 34% and are increasing [2]. Accumulating evidence suggests that MO predisposes offspring to major risk factors, including obesity, insulin resistance, and cardiovascular dysfunction [3, 4, 5]. Cardiovascular disease is the number one cause of death globally, with more than 17 million deaths reported annually [6]. MO induces cardiac ventricular hypertrophy, inflammation, and cardiomyocyte contractile dysfunction in their offspring [7, 8].

One feature of cardiovascular dysfunction induced by MO is cardiac remodeling; an accumulation of extra cellular matrix proteins such as collagen (fibrosis) between muscle fibers and vessels [9]. Fibrosis in heart tissue is regulated by metalloproteinases and their inhibitors [10] and may diminish cardiomyocyte contractile function and eventually lead to heart failure [11]. Studies have shown that obesity may lead to fibrosis in the hearts of rats and mice [12]. Data from our lab showed that MO in sheep induces fibrosis in the myocardium of their fetuses [13] leading to increased ventricular weight and wall thickness, as well as the inability to sustain high work levels *in vitro* [14]. However, whether MO programs increased fibrosis in the hearts of their adult offspring has not been evaluated.

Overnutrition during pregnancy expands lipid deposition around the fetal heart and leads to macrophage infiltration and up-regulation of pro-inflammatory cytokines which in turn induce cardiovascular dysfunction and metabolic syndrome phenotypes in the offspring [15, 16, 17]. Fibrosis is usually accompanied by a low-grade inflammatory response [18]. Studies have shown that MO induces inflammation [19, 20, 21] which has been demonstrated as a cause for myocardial fibrosis and hypertension in rats [22]. MO-induced myocardial fibrosis in the fetus was also associated with inflammation and upregulation of transforming growth factor-β (TGF-β) /p38 signaling pathway [13]. TGF-β promotes pathological fibrosis via activation of the Smad signaling pathway (NF-κB) [14]. MO is associated with elevated plasma cortisol in mid and late gestation fetuses and in their adult offspring [23, 24]. It is noteworthy that fibrosis and up-regulation of proinflammatory cytokines is also seen in the hearts of adult sheep offspring of nutrient restricted mothers [25]. Chronic exposure to cortisol can lead to hypertension and cardiovascular morbidity and mortality which are the main consequences of Cushing’s syndrome [26]. Glucocorticoids play important roles in diverse physiological processes such as; glucose metabolism, immune system function, and fetal heart development [27, 28]. Further, glucocorticoids have been widely used as anti-inflammatory drugs that can reduce morbidity and mortality in premature babies. However, long-term treatment with these steroids results in many complications including cardiovascular disorders [29]. Cortisol induces its action by binding to nuclear glucocorticoid receptors (GR) and mineralocorticoid receptors (MR), which act as transcription factors when bound to a ligand. Both GR and MR are widely expressed in the heart whereby continued activation of GR and MR by chronic exposure to glucocorticoids and can lead to fibrosis, hypertension, and diastolic dysfunction [30, 31]. The role of MR in inducing cardiac diastolic dysfunction, hypertension, and cardiac remodeling has been reported [32, 33].

The aim of this study was to investigate the effect of chronically elevated cortisol induced by MO on the heart of their adult male offspring. Based on the pathological changes seen in these animals, our hypothesis is that the chronic exposure to elevated cortisol, which is initiated *in utero* and later induced by *ad libitum* feeding in adulthood may have adverse effects in the cardiovascular system of adult offspring potentially through the stimulation of increased GR activity.

## Materials and methods

### Animals

Animals and procedures used in this project were approved by the University of Wyoming Animal Care and Use Committee. Ewes were assigned randomly to a control (CON, 100% of NRC recommendations) or obese (MO, 150% of NRC) diet from two months before conception to term. Singleton male lambs (n = 6/maternal dietary group) were penned together and fed only to NRC recommendations from weaning until 19 months of age, then placed on a 12-week *ad libitum* feeding challenge. At the end of the feeding challenge, 20 ml of blood was drawn by venipuncture, then immediately centrifuged at 2,500 RPM for 15 minutes at 4°C and plasma stored at -80°C until analyzed. Male lambs were euthanized with an overdose of sodium pentobarbital (Beuthanasia-D Special; Schering-Plough Animal Health, Union, NJ) and the heart was quickly removed. The whole heart was weighed and left and right ventricular free wall dissected and weighed. Digital calipers (Absolute, Digimatic Caliper,Mitutoyo) were used to measure ventricular thicknesses and recorded at 3 random sites across each ventricular wall excluding the papillary muscles and values averaged as previously described [34]. Samples of myocardium were then collected from the left and right ventricular free walls, snap frozen in liquid nitrogen, and stored at –80°C until utilized for collagen analyses and mRNA and protein quantification.

### Cortisol assay

Plasma cortisol was determined in duplicate as previously described [35], using a commercial cortisol radioimmunoassay (RIA) kit with a sensitivity of 0.5 μg/dL (Siemens Healthcare Diagnostics, Deerfield, IL, USA). All cortisol measurements were completed in a single assay and the intra-assay coefficient of variation (CV) was 4.5%.

### Collagen and pyridinoline cross-linking determinations

Approximately 100 mg of heart tissue was ground and dried in a convection oven at 60°C, thereafter, the sample was reweighed and hydrolyzed in 6 N HCl at 105°C for 16 h. After HCL digestion, sample was neutralized with NaOH. An aliquot was saved for pyridinoline cross-linking analysis. Collagen concentration (mg/g dry muscle weight) was calculated based on hydroxyproline equivalent as published previously in our laboratory [13]. Oxidation of hydroxyproline with 4-(Dimethylamino) benzaldehyde (DMAB) results in a colorimetric (560 nm) product, proportional to the hydroxyproline concentration present in the sample. The intra-assay coefficient of variation (CV) was 13.3%. Pyridinoline crosslinks concentration in the sample was measured with Metra Serum PYD EIA kits (Quidel, San Diego, CA) following the company’s protocol. The pyridinoline concentration was expressed as nmol/mg of dry muscle weight and the intra-assay coefficient of variation (CV) was 3.29%.

### RNA extraction, cDNA synthesis, and quantitative PCR analysis

Total RNA from 150mg of each sample of myocardial tissue was extracted using Trizol reagent (ThermoFisher, Waltham, MA), then treated with DNase I to digest DNA from RNA and then purified by RNeasy mini column (QIAGEN Inc. Valencia, CA) to obtain high-quality RNA from the tissue according to the manufacturer’s instructions. The quality of extracted RNA were determined spectrophotometrically by measuring the OD260/280 ratio in a pH neutral buffer, an OD260/280 of 2.0 which indicates good quality RNA were achieved. RNA integrity was evaluated by gel electrophoresis. Two μg of RNA was reverse transcribed into cDNA using a cDNA synthesis kit (QuantiTect Reverse Transcription Kit, QIAGEN, Valencia, CA) according to the manufacturer’s instructions. Quantitative RT-PCR was performed using a Bio-Rad IQ5 Real-time-PCR Reaction System (Bio-Rad Laboratories Inc., Hercules, CA). Gene names and sequences of the primers used are listed in **Table 1.**

**Table 1:** Primers sequences used for real-time-PCR.

Primer	Forward sequence	Reverse sequence
**TGF-β1**	5’-CACGTGGAGCTGTACCAGAA-3’	5’-GGCGAAAGCCTTCTATTTCC-3’
**TNFα**	5’-TTCAGGAGGTCAAGGTGTCC-3’	5’-GCGACAAATCAGTCACCAAA-3’
**IL-6**	5’-GTTCAATCAGGCGATTTGCT-3’	5’-CAGCATGTCAGTGTGTGTG G-3’
**CD14**	5’-CTCAGCGTGCTTGATCTCAG-3’	5’-AAGGGATTTCCGTCCAGAGT-3’
**CD68**	5’-CAGGGGACAGGGAATGACT-3’	5’-CCAAGTGGTTGTTCTGTGG-3’
**IL-18**	5’-ATGGCGAAGACCTGGAATC-3’	5’-CAGGTTGATTTCCCTGGCTA-3’
**TLR4**	5’-TGCTGGCTGCAAAAAGTATG-3’	5’-CCCTGTAGTGAAGGCAGAGC-3’
**GR**	5’-AAGTCATTGAACCCGAGGTG-3’	5’- TGCAGCAGAGTCATTTGGTC-3’
**GAPDH**	5’-ACTGGCAAAGTGGACATCGT-3’	5’-CCAGCATCACCCCACTTGAT-3’

PCR conditions used are as follows: 20 s at 95°C, 20 s at 56°C and 20 s at 72°C for 35 cycles. The expression of each gene was determined after normalization with an expression of the house keeping gene GAPDH. This is widely used in qPCR as housekeeping gene. Although it is a metabolic enzyme, in our study no treatment effects on its expression was observed in the heart between the groups. The relative expression level (in fold) was measured by using the 2^−(ΔΔ^*Ct*^)^ method [36].

### Protein extraction and western blot analysis

Heart samples were pulverized in liquid nitrogen, 100 mg of each sample were homogenized using a polytron homogenizer (Kinematica, Bohemia, NY) with 1ml of 1x Laemmle buffer pH 6.8 and 1% protease inhibitor cocktail, (Promega, Madison.WI). Homogenates were then centrifuged and the supernatants boiled at 95°C for 5 minutes. Proteins extracts were collected after a 10-min centrifugation at 12,000 × g. Protein concentrations were determined using a NanoDrop 2000C spectrophotometer (Thermo Scientific, Wilmington, DE). Western blot assay was performed as previously published from our laboratory [37]. Briefly, ~50 μg of protein in loading buffer was separated by 10% SDS-PAGE gel and transferred to a nitrocellulose membrane. The membranes were blocked with (5% nonfat dry milk in Tris-buffered saline-Tween 20 buffer containing 150 mM NaCl, 10 mM Tris, pH 7.6, and 0.05% Tween 20) for 1 hr at room temperature. Membranes were probed with primary antibodies against the following proteins: GR (Ab2768), MR (Ab2774), 11β-hydroxysteroid dehydrogenase type 1 (11β-HSD1) (Ab39364), hexose-6-phosphate dehydrogenase (H6PD) (Ab 84353), TNF-α (Ab106606), CD 68(Ab63896), purchased from Abcam Inc. (Cambridge, MA), and TGF-β1 (3711), purchased from Cell Signaling (Danvers, MA). All antibodies were diluted in 5% (wt/vol) nonfat milk, at 1:500 overnight at 4°C. Probed membranes were further incubated with a secondary antibody conjugated with horseradish peroxidase at 1:3,000; concentration was diluted in 2% (wt/vol) nonfat milk for 60 min at room temperature. Membranes were visualized using enhanced chemiluminescence (ECL) western blotting reagents and exposed to an X-ray film (MR; Kodak, Rochester, NY). The density of bands was quantified by using ImageJ. Band density was normalized according to the density of β-actin content (anti-β-actin, cat. no. 4790; Cell Signaling Technology, Danvers, MA).

### Statistical analysis

Data were analyzed using the MIXED procedure of SAS (SAS Institute, Cary, NC) with treatment as main effect. Statistical significance (p < 0.05) was estimated by Student’s unpaired t-test. Prism (GraphPad Software Inc, La Jolla, CA) was used to make the graphs.

Data are presented as least square (LS) means ± SEM, and differences are considered significant at P ≤ 0.05, with a tendency at P < 0.1.

## Results

### Plasma cortisol and myocardial phenotype

**Fig 1:**
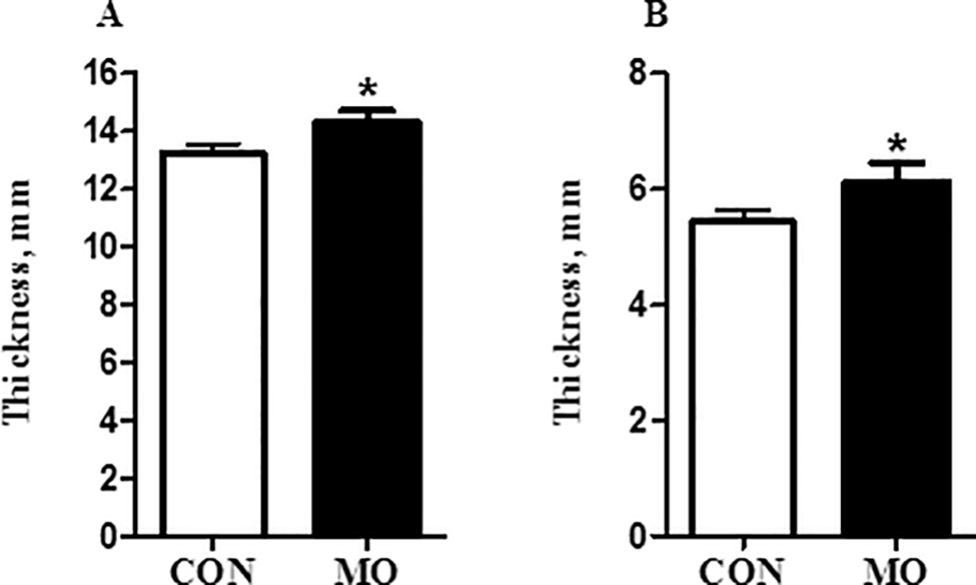
Left ventricular wall thickness (panel A) and right ventricular wall thickness (panel B) of adult CON offspring (open bars) and adult MO offspring (solid bars). Data are presented as *LS Means ± SEM differ, (P < 0.05); n = 6/group, and analyzed by Student's t test.

**Fig 2:**
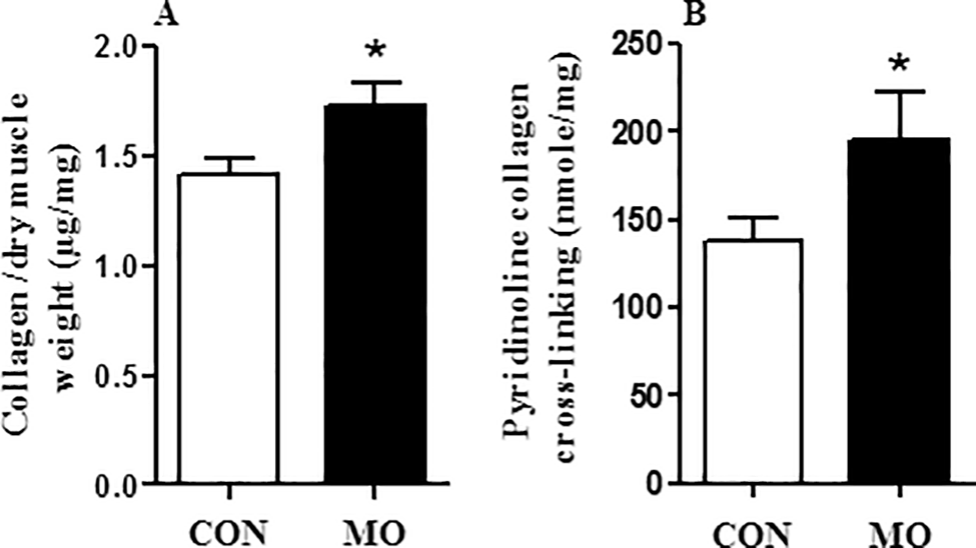
Collagen concentrations (panel A) and collagen crosslinking (panel B), in ventricular tissue of adult offspring. Open bars are CON offspring and solid bars are MO offspring. *LS Means ± SEM differ, (P < 0.05); n = 6/group. Data are analyzed by Student's t test.

At necropsy, plasma cortisol concentrations were higher in MO offspring than in CON offspring (6.16 ± 1.01 versus 3.3 ± 0.9 ng/ml, respectively; (P < 0.05). Both the left and right ventricular thickness were greater (P < 0.05) in adult male MO offspring than CON offspring; **Fig 1A** and **1B** respectively. When both the left and right ventricular thickness corrected for heart weight, the results showed no significant differences (P > 0.05) in adult male MO offspring than CON offspring (0.032± 0.0014 versus 0.03± 0.0014 mm) and (0.0135± 0.0006 versus 0.012± 0.0006 mm, respectively. Collagen concentration in ventricular heart tissue was measured by determining hydroxyproline equivalents in the sample. Collagen content was greater in MO offspring than CON offspring (1.73 ± 0.10 versus 1.42 ± 0.07 μg/mg, respectively; (P < 0.05) **Fig 2A**. Collagen crosslinking as measured by determining pyridinoline concentration in the sample was also greater (P < 0.05) in the hearts of MO offspring compared to CON offspring; **Fig 2B**.

### Myocardial mRNA and protein expression

**Fig 3:**
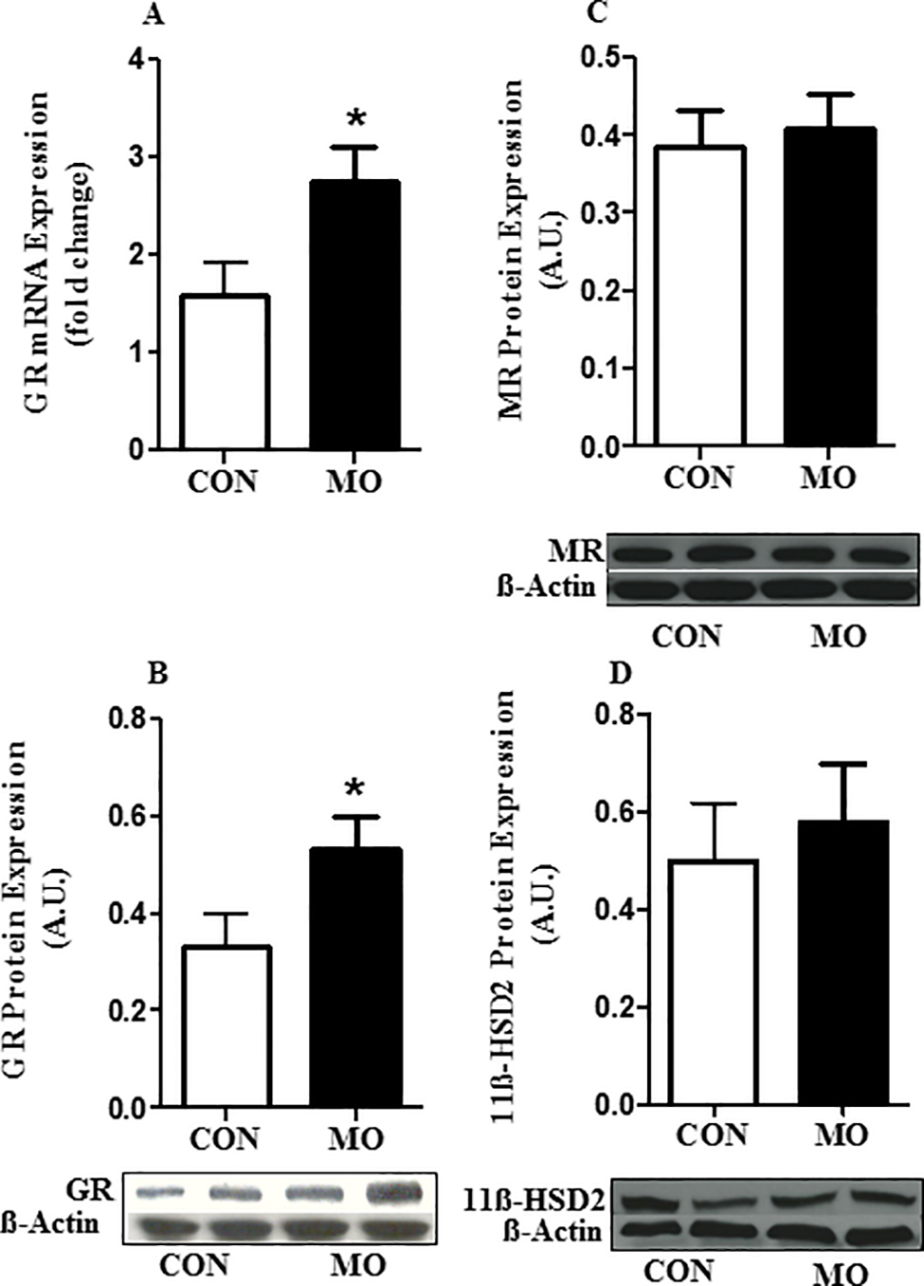
mRNA expression of GR (panel A), protein expression of GR (panel B), protein expression of MR (panel C), and protein expression of 11ß-HSD2 (panel D) in the myocardium of CON (open bars) and MO (solid bars) adult male offspring. *LS Means ± SEM differ, (P < 0.05). #LS Means ± SEM differ, (P < 0.1); n = 6/group. Data are analyzed by Student's t test.

Cardiac GR mRNA and protein expression were greater in MO than CON offspring (P < 0.05) (**Fig 3A and 3B**). In contrast, cardiac MR protein expression was similar in CON and MO offspring **(Fig 3C).** No protein expression was detected for the enzyme 11β-HSD1 or its cofactor H6PD. However, no significant differences were observed in cardiac protein expression of 11β-HSD2 between the groups **(Fig 3D)**.

### Myocardial expression of proinflammatory cytokines

**Fig 4:**
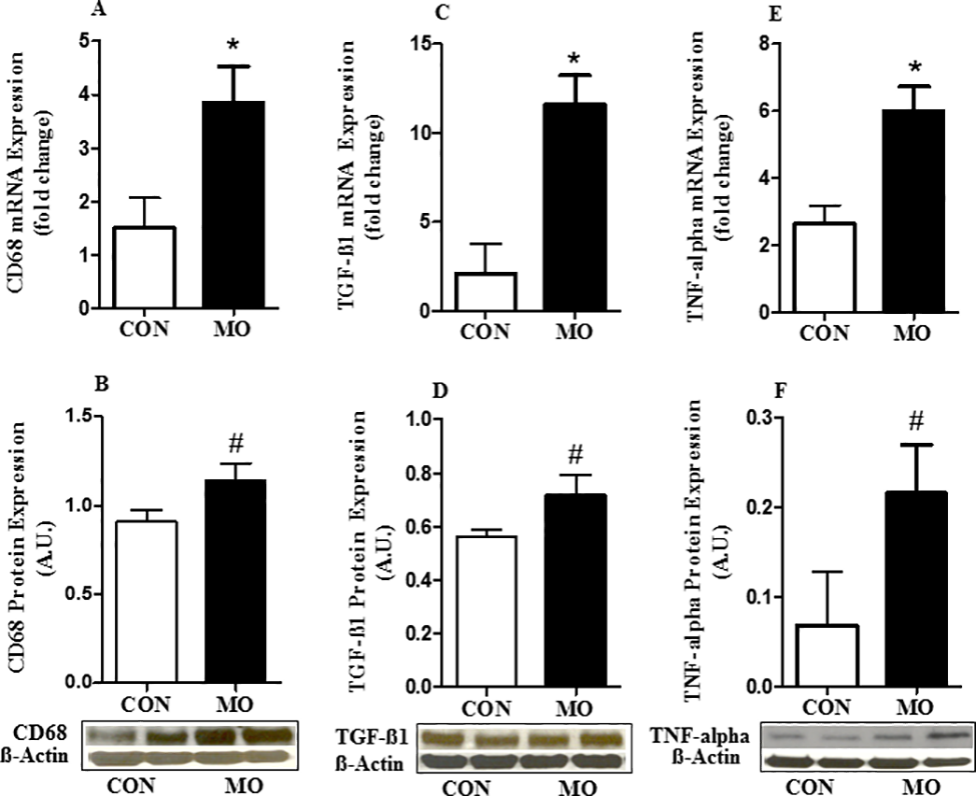
mRNA expression of CD68 (panel A), protein expression of CD68 (panel B), mRNA gene expression of TGF-ß1 (panel C), protein expression of TGF-ß1 (panel D), mRNA gene expression of TNF-alpha (panel E), and protein expression of TNF-alpha (panel F), in the myocardium of CON (open bars) and MO (solid bars) adult male offspring. *LS Means ± SEM differ, (P < 0.05). ^#^LS Means ± SEM tended to differ, (P < 0.1); n = 6/group. Data are analyzed by Student's t test.

**Fig 5:**
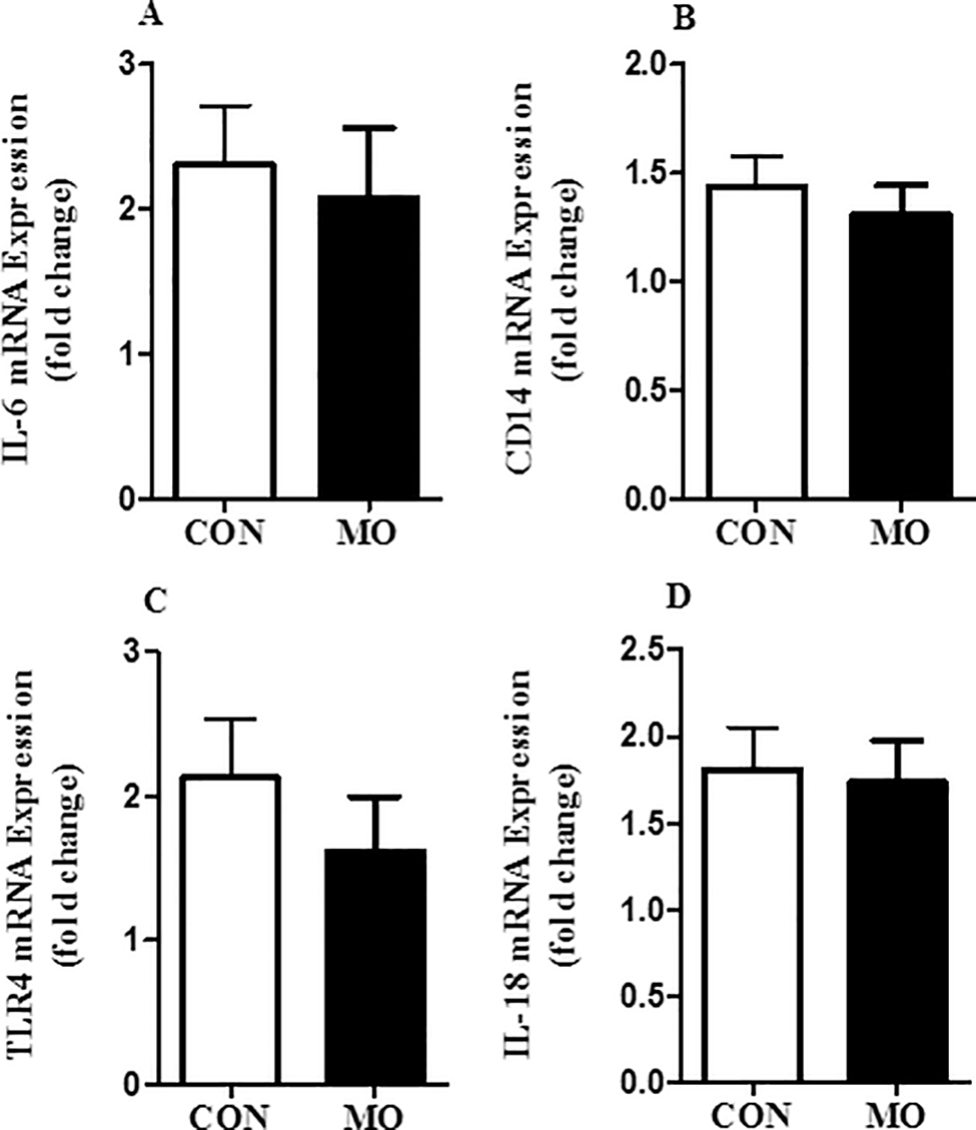
mRNA expression of IL-6 (panel A), CD14 (panel B), TLR4 (panel C), and IL-18 (panel D), in myocardial samples of CON (open bars) and MO (solid bars) offspring; n = 6/group. Data are presented as *LS Means ± SEM differ, (P < 0.05); n = 6/group, and analyzed by Student's t test.

CD68 is an important marker for tissue macrophages; myocardial mRNA expression of CD68 was greater in MO than CON offspring (P < 0.05) **(Fig 4A)**. Further, protein expression of CD68 tended to be higher in cardiac tissue of MO vs. CON offspring (P < 0.10) **(Fig 4B)**.Cardiac mRNA expression of TGF-β1 was greater (P < 0.05) in MO vs. CON offspring **(Fig 4C)**. Protein expression of TGF-β1 also tended to be greater in MO vs. CON offspring (P < 0.10) (**Fig 4D)**.Cardiac mRNA expression of TNF-α was greater (P < 0.05) in MO vs. CON offspring **(Fig 4E)**. Protein expression of TNF-α also tended to be increased in MO vs. CON offspring (P < 0.10) (**Fig 4F)**. In contrast, there were no differences (P > 0.10) found in cardiac mRNA of IL-6, CD14, TLR4, and IL-18 cytokine gene expression **(Fig 5)**.

## Discussion

The 12 weeks ad libitum feeding challenge was incorporated in our protocol because our previous findings showed no differences in growth rate or adiposity when both MO and CON offspring are maintained together from weaning to maturity and only fed to control diet. Therefore, a second exposure to stressful condition (in this case ad libitum feeding challenge) is required during adulthood to elicit phenotypical differences in postnatal metabolism and body composition based on the two hit hypothesis [38].

In the present study, we have demonstrated that MO leads to programing of myocardial inflammation and fibrosis in adult male offspring. These pathologic changes were associated with elevated plasma cortisol levels and increased expression of GR in the heart. The results further demonstrate an increased collagen and collagen cross-linking and up-regulation of the proinflammatory cytokines CD68, TGF-β1, and TNF-α in myocardial tissue.

These data suggest that GR activation by cortisol may contribute to the associated pathologic changes seen in the heart muscle. Plasma cortisol concentration is determined by the activity of the HPA axis, MO induces an increase in hypothalamic-pituitary stimulation of cortisol synthesis and secretion by the adrenal of their adult offspring [23]. However, cortisol can also be generated in peripheral tissues such as liver and adipose tissues by the enzyme 11β-HSD1 which are known to convert cortisone to cortisol leading to local tissue cortisol excess [39]. We recently reported that cortisol concentrations are increased in the liver and blood of fetal and adult male MO offspring through increased 11β-HSD1 and its co-factor H6PDH in liver tissue [40, 41]. The present study shows that11β-HSD1 and H6PDH are very low in adult heart tissue. Previous studies have reported that the expression of 11β-HSD1 in the heart is low and that its presence may exacerbate pathological heart changes [42].

Thus the pathological changes seen in the heart of adult MO offspring may be due to elevated levels of circulatory plasma cortisol rather than the local production of cortisol within the heart itself. The activity 11β-HSD2 in the heart is also usually very low in physiological conditions, however, this may not be the case during pathological conditions such as hypoxia where both the activity and expression of 11β-HSD2 are increased [42], our results showed the presence of 11β-HSD2 in myocardial tissue but with no significant differences between the groups. The 11β-HSD2 enzyme is known to convert the active cortisol to inactive cortisone [42].Therefore, 11β-HSD2 presence in the heart may promote protective effect to the heart [43]. Both GR and MR are expressed in cardiomyocytes [44] and both receptors are able to bind to cortisol. Our results showed increased expression of GR but not MR in heart tissue of MO offspring. Sheep studies aimed to mimic maternal stress showed that fetal short exposure to cortisol (10 days) at 130 days of gestation showed increased left ventricular thickness and proliferation and apoptosis in the heart. These proliferative and apoptotic effects of cortisol were induced by MR and GR respectively, [45]. Apoptosis is gene regulated programmed cell death and can lead to fibrosis in different types of tissues, including the heart [46]. Sufficient cortisol concentration is required for fetal heart maturation by GR- cortisol action, disruption of GR signaling in cardiomyocytes of mice resulted in an immature dysfunctional heart [27]. Obese rats treated with the GR antagonist Mifepristone (RU486) had reduced left ventricular fibrosis, diastolic dysfunction, cardiac oxidative stress, and inflammation [47]. Moreover, rat cardiomyocytes, treated *in vitro* with Dexamethasone, led to increased cell size and up-regulation of the hypertrophic markers, and these changes were abolished by RU486 and GR gene knockdown [48]. However, blocking the activity of MR with the antagonist Eplerenone or MR gene knockdown did not inhibit Dexamethasone mediated cardiomyocyte hypertrophy in rats [48]. RU486 is a competitive antagonist, and binds to GR with 3- fold higher affinity than Dexamethasone and 18-fold higher affinity than cortisol [49]. Eplerenone is an aldosterone-MR antagonist widely used for hypertension and heart failure treatment [50]. Although there was no difference in the protein expression of MR in OB compared to CON offspring in our study, there is cumulating evidence linking MR-aldosterone activation to hypertension and heart failure [51].

Our study demonstrated increased collagen and collagen cross-linking and up-regulation of CD68, TGF-β1, and TNF-α in myocardial tissue of MO adult offspring. Previous findings in several animal models have demonstrated that MO led to inflammation and fibrosis in the hearts of offspring [13, 52, 53]. In humans, MO is linked to premature death in adult offspring due to cardiovascular disease, pregnant MO women have increased circulation of inflammatory cytokines, fatty acids, and insulin resistance, these may induces hypertension and myocardial fibrosis in adult offspring as seen in other animal models [54]. Further, accumulation of adipose tissue can induce production of proinflammatory cytokines [55, 56] leading to lipotoxicity which can alter cardiovascular function in adult offspring [57]. Spencer et al., [58] reported that increased recruitment of inflammatory cytokines and collagen accumulation in human adipose tissue increases with body mass index (BMI). Fibrosis is trigged by inflammation [59] and regulated by the TGF-β /p38 signaling pathway [60], which may negatively alter cardiac function [11].

The present findings are consistent with our previous observations showing that MO upregulates TGF-β expression leading to fibrosis in the fetal heart [13] and the enlargement of the left and right ventricular wall thickness during mid gestation [61]. Moreover, overexpression of TNF-α in transgenic mice led to myocarditis, production of nitric oxide, and heart failure [62, 63]. CD 68 is a marker of macrophage infiltration and its presence, along with TNF-α, in heart tissue during chronic heart failure in humans, has been reported [64]. No significant differences in gene expression of IL-6, IL-18, CD14, and TLR4 cytokines in the present study, although previous data from our lab showed that MO induced inflammation and cardiac morphometry in the fetal heart, and alteration of the expression of IL-6, IL-18, CD14, and TLR4 [65]. It appears that these alterations do not persist in heart of adult MO offspring. This study supports our hypothesis that the MO-induced cardiac inflammation and fibrosis seen during fetal development persists in the myocardium of adult offspring after *ad libitum* feeding challenge. Since most of maternal insults upon the fetus are associated with increased levels of glucocorticoids, thus, these finding have broader implications for understanding how exposure to elevated cortisol concentration affects offspring health, especially, on the cardiovascular development. Our findings further suggest a potential role of increased myocardial GR in inducing the observed pathological changes seen in the heart. Future functional studies might be needed to confirm whether these changes seen in the heart are mediated through GR by blocking the activity of GR using GR antagonists or inhibitors.

In conclusion, these data demonstrate that MO during ovine pregnancy elevates blood cortisol concentrations from the adrenal or other body tissues (i.e. liver) and may predispose adult male offspring to cardiac dysfunction due to up-regulation of proinflammatory cytokines and fibrosis.

## Supporting information

S1 Table (PDF)
